# McHenry County Medical Society

**Published:** 1852-11

**Authors:** E. G. Mygatt, C. B. Durfee

**Affiliations:** President; Secretary


*McHenry Co. Medical Society.*

At a meeting of the physicians of McHenry County, held pursuant to notice, in Woodstock, July 24th, 1852, the following Constitution was presented and adopted.

CONSTITUTION.

Art. 1. The name of this society shall be the McHenry County Medical Society.

Art. 2. The object of this society shall be to elevate the standard of the profession, by the diffusion of medical knowledge, and to promote unanimity of feeling, and concert of action, among the members thereof.

Art. 3. Any regular practitioner of medicine, of good moral character, residing in the county, may become a member of this society, by passing a satisfactory examination before the Board of Censors, and by a two-third vote of the members present at any regular meeting.

Art. 4. The physicians present at the adoption of this Constitution, shall be considered members of this Society.

Art. 5. The officers of this society shall be a President, Vice President, Secretary, (who shall act as Treasurer,) and three Censors, who shall be elected by a majority of the members present at each annual meeting.

Art. 6. The annual meeting of this Society shall be held at Woodstock, on the first Tuesday in January of each year.

Art. 7. This Constitution may be altered or amended by a two-third vote of the members present at any regular meeting of this Society.

The Society thus organized, consists of the following members:

| | |
|---|---|
| Dr. M. A. Smith, | Dr. Wm. B. Hart, |
| " E. G. Mygatt, | " M. Baldwin, |
| " M. F. Irwin, | " C. B. Durfee, |
| " W. S. Allaben, | " O. S. Johnson, |
| " Edward Bennett, | " C. S. White, |
| " R. R. Stone, | " W. F. Colman, |
| " H. T. Brown, | |

The following officers were elected for the ensuing year:

E. G. MYGATT, President,

M. A. SMITH, Vice President,

C. B. DURFEE, Secretary and Treasurer.

WM. B. HART,<br>O. S. JOHNSON,<br>M. F. IRWIN, } Censors.

Resolved, That the Woodstock Democrat, and the North-Western Medical and Surgical Journal, be furnished a copy of the above for publication.

E. G. MYGATT, President.

C. B. DURFEE, Secretary.

